# Chinese medicine for residual symptoms of COVID-19 recovered patients (long COVID)—A double-blind, randomized, and placebo-controlled clinical trial protocol

**DOI:** 10.3389/fmed.2022.990639

**Published:** 2023-01-04

**Authors:** Chi Him Sum, Jessica Yuet Ling Ching, Tianhe Song, Pui Kuan Cheong, Cho Wing Lo, Mei Kwan Lai, Chon Pin Chia, Kam Leung Chan, Wing Yan Mak, Ka Chun Leung, Sin Bond Leung, Hongwei Zhang, Zhixiu Lin

**Affiliations:** ^1^Hong Kong Institute of Integrative Medicine, The Chinese University of Hong Kong, Hong Kong, Hong Kong SAR, China; ^2^Department of Medicine and Therapeutics, Faculty of Medicine, The Chinese University of Hong Kong, Hong Kong, Hong Kong SAR, China; ^3^School of Chinese Medicine, Faculty of Medicine, The Chinese University of Hong Kong, Hong Kong, Hong Kong SAR, China; ^4^Institute of Digestive Disease, The Chinese University of Hong Kong, Hong Kong, Hong Kong SAR, China

**Keywords:** Chinese medicine, alternative and complementary medicine, long COVID-19, randomized controlled trial, protocol

## Abstract

**Introduction:**

Coronavirus disease 2019 (COVID-19) is the current global pandemic of which residual symptoms exhibited by post-acute, rehabilitating patients include fatigue, dyspnoea, and insomnia. Chinese medicine (CM) has been widely used in China to treat different stages of COVID-19. While there are a significant number of clinical studies suggesting its efficacy and safety in its use during acute stage, there are very few randomized controlled trials focusing on the rehabilitation stage. Liujunzhi Decoction and Shashen Maidong Decoction are frequently recommended by official clinical guidelines in China to treat COVID-19 patients in rehabilitation stage. This double-blind, randomized, placebo controlled study aims to evaluate the efficacy and safety of the combination of the two formulae [named “COVID-19 Rehab Formula (CRF)”] in treating COVID-19 residual symptoms (long COVID).

**Methods:**

Eligible subjects will be randomly divided into treatment group and control group in 1:1 ratio. Treatment group will receive CRF along with certain pre-defined CM according to symptoms for 8 weeks, while control group will receive equivalent packs of placebo for 8 weeks. Data in terms of Fatigue Severity Score (FSS), self-reported COVID-19 long term symptom assessment, the modified British Medical Research Council (mMRC) Dyspnoea Scale, EuroQol Five-Dimension Five-Level (EQ-5D-5L) Questionnaire, pulmonary function test and adverse events will be collected and analyzed by SPSS 24. Blood test on liver and renal functions will also be conducted as safety measures.

**Conclusion:**

This study will evaluate the efficacy and safety of CRF in the treatment COVID-19 residual symptoms in a scientifically rigorous design.

**Clinical trial registration:**

[ClinicalTrials.gov], identifier [NCT04924881].

## 1. Background

The first identification in patients with severe pneumonia was in Wuhan province, China in November 2019. A novel coronavirus was identified as the cause by Chinese authorities on 7 January 2020 and was temporarily named “2019-nCoV.” Coronaviruses (CoV) are a large family of viruses that cause illness ranging from the common cold to more severe diseases. A novel coronavirus (nCoV) is a new strain that has not been previously identified in humans. The new virus was subsequently named the “COVID-19 virus.” COVID-19 has spread rapidly and now affects all over the world. This is the greatest pandemic of modern times and has been declared a Public Health Emergency of International Concern by the WHO Director-General (1). On 11 March 2020, coronavirus disease 2019 (COVID-19) was declared a global pandemic by the World Health Organization (WHO). As of 5 June 2022, over 529 million COVID-19 confirmed cases were reported worldwide, with more than 6 million related death (2).

Most of the infected people will develop mild to moderate illness, for example fever, cough, tiredness and joint pain etc. For some older people, and those with comorbidities like cardiovascular disease, diabetes, chronic respiratory disease, and malignancy are more likely to develop serious illness (2).

Up to now, most frequently used therapies are corticosteroids, antiviral agents, antiviral/immunomodulatory drugs, serotherapy, anticoagulant and inflammation inhibitors (3). The medium and long-term problems experienced by survivors of COVID-19 after discharge from hospital include fatigue, breathlessness and joint pain (4). Studies reported that different kinds of residual symptoms were left after patient discharged from the hospital. More than eight-seven percent reported a persistence of at least one symptom, particularly fatigue and dyspnoea 4 weeks after post-discharge (5). Overall, more than fifty four percent of all female patients reported moderate or severe fatigue, compared to 30 percent of male patients. Moderate or severe dyspnoea was also more often reported by females than males when they were under intensive care (53.8 vs. 21.1%) but the proportions were similar in those who stayed in ordinary ward (24.2 and 20.0%) (4). Besides, thirteen percent of patients had gastrointestinal symptoms such as diarrhea, vomiting, loss of appetite, etc. A cohort study showed that some COVID-19 recovered patients suffered from the above symptom up to 6 months or more (6). In Hong Kong, our data showed that 26.7 suffered fatigue, 20% suffered dyspnoea and 23.3% insomnia which are residual symptoms 6 months after discharged from the hospital of our COVID-19 recovered patients (unpublished data).

Traditional Chinese Medicine (TCM) has a long history and played an important role in the prevention and treatment of several epidemic diseases. During SARS epidemic in 2003, the treatment of TCM has achieved remarkable therapeutic effect. Some recent studies have demonstrated that among the limited therapeutics, TCM plays an important role in halting the progress of the disease and promoting the recovery of patients in the absence of vaccines or targeted drugs (7). As early as of 17 February 2020, total number of confirmed COVID-19 cases treated by TCM had already reached 60,107 in China (8).

Many different types of TCM have been proposed to treat COVID-19, of which the most famous are “three medicines and three formulae”: Jinhua Qinggan Granule, Lianhua Qingwen Capsule/Granule, Xuebijing Injection; Lung Cleansing and Detoxifying Decoction, HuaShiBaiDu Formula and XuanFeiBaiDu Formula, which are mainly used for active COVID cases of different severity. Clinical and pharmacological studies have suggested their efficacy, safety, and possible mechanisms in treating different stages of COVID-19, either used along with conventional treatment or independently (9–14).

For residual symptoms seen in COVID recovered patients, such as fatigue, dyspnoea and insomnia, there are also studies suggesting that TCM may be helpful. For example, a meta-analysis of 11 studies has shown that using Chinese medicine interventions together with conventional treatment is more effective than using the conventional treatment alone in treating chronic fatigue syndrome (15). There are also meta-analyses that have shown similar conclusions for idiopathic pulmonary fibrosis (16) and insomnia (17).

Liujunzhi Decoction (LJZD) and Shashen Maidong Decoction (SSMDD) are classic Chinese medicine formulae that have been used in China for hundreds of years. LJZD is composed of six herbs, namely, Ginseng Radix Et Rhizoma, Atractylodis Macrocephalae Rhizoma, Poria, Glycyrrhizae Radix Et Rhizoma Praeparata Cum Melle, Pinelliae Rhizoma Praeparatum and Citri Reticulatae Pericarpium, while SSMDD of seven: Adenophorae Radix, Polygonati Odorati Rhizoma, Glycyrrhizae Radix Et Rhizoma, Mori Folium, Ophiopogonis Radix, Lablab Semen Album, and Trichosanthis Radix. Their combination is proposed as the intervention of this trial because, from the perspective of Chinese medicine, the residual symptoms of COVID-19 can often be seen as the manifestation of “lung-spleen qi deficiency” or “dual deficiency of qi and yin” (18, 19). LJZD and SSMDD are two of the classic formulae used to treat these two pathologies, respectively. In fact, among the 33 official COVID-19 clinical guidelines published in mainland China, 10 have recommended the use of LJZD, and 6 recommended SSMDD (or their modified versions) in the recovery stage of COVID-19. Modified LJZD and SSMDD are the two most frequently recommended formulae (20).

To date, there are very few clinical studies of using TCM to treat the residual symptoms of COVID-19 recovered patients. A multi-center observational study in Hong Kong has shown that individualized TCM treatments could facilitate resolution of clinical symptoms (including fatigue, cough, shortness of breath, post-meal fullness, and loose stool), improve lung functions, and lead to healthier CM body constitutions in COVID recovered patients (21). A randomized controlled trial comparing a Chinese medicine (Qingjin Yiqi granules) combined with standard rehabilitation treatments (SRTs) against SRTs alone has also shown that the CM granules could assist reduction in fatigue and breathlessness (22). However, there is still a lack of double-blind, placebo-controlled randomized controlled trials on the subject—a void this study aims to fill.

## 2. Hypothesis

The residual symptoms of COVID-19 recovered patients can be improved after taking Chinese medicine.

## 3. Objectives

To evaluate the efficacy of using COVID Rehab Formula “CRF” (LJZD and SSMDD with variations) on the residual symptoms of COVID-19 recovered subjects.

## 4. Study outcomes

### 4.1. Primary outcome

Improvement of residual COVID-19 symptoms of fatigue using Fatigue Severity Score (FSS) at week 8.

### 4.2. Secondary outcomes

1. Self-reported COVID-19 Long Term Symptom Assessment at week 8.
2. Improvement of fatigue using Fatigue Severity Score (FSS) at week 12.
3. Change of modified British Medical Research Council (mMRC) dyspnoea scale to measure the improvement of dyspnoea at week 8 and 12.
4. Change of EuroQol five-dimension five-level (EQ-5D-5L) questionnaire and it’s Visual Analogue Scale (VAS) to measure the quality of life at week 8 and 12.
5. Improvement of pulmonary function FEV1, FVC, and FEV1/FVC ratio at week 8.
6. Adverse events related to study treatment.

## 5. Study design

This is a double-blind, randomized, placebo-controlled superiority trial. Eligible subjects will receive either “CRF” granules or placebo granules for 8 weeks followed by a post-treatment visits at week 12.

## 6. Study population

COVID-19 recovered patient with a confirmed diagnosis of SARS-Cov-2 infection using the PCR or rapid antigen test according to the standard of Center for Health Protection, Department of Health, Hong Kong and released from isolation^1^ will be screened for the following eligibility criteria. Study subjects will be recruited from the following clinics/Chinese medicine centers: (1) The CUHK Chinese Medicine Specialty Clinic and Teaching and Research Center on CUHK campus (CUHK-CMSCTRC); (2) The two Integrative Medical Centers, Hong Kong Institute of Integrative Medicine at Shatin and Wan Chai. Moreover, patients following up for residual symptoms at the medical clinics at Prince of Wales Hospital will be provided our study information; interested patients can go to our Integrative Medical Center located at the same hospital. Advertisements in the poster on the clinic and internet platforms, such as Facebook, emails, and website will be made to facilitate community recruitment. Besides, we will publish articles in local newspapers and magazines as well as organize health promotion talks to augment the subject recruitment process.

### 6.1. Inclusion criteria

• Aged above 18.
• Have fatigue and one more residual symptoms (e.g., dyspnoea, sleep disturbance, cough, loose stool, abdominal distension, loss of appetite, dizziness, etc.) at least 5 weeks after discharge.
• Patients are diagnosed with “lung-spleen qi deficiency” and/or“dual deficiency of qi and yin” by a Chinese Medicine Practitioner.
• Voluntary written consent.

### 6.2. Exclusion criteria

• Still being SARS-CoV-2 positive.
• Known severe medical conditions, such as cardiovascular, liver or renal dysfunction, diabetes mellitus, cancers, cerebrovascular diseases, and blood system diseases.
• Impaired hematological profile and liver/renal function.
• No concomitant non-steroidal anti-inflammatory drugs (NSAIDs), steroids, antibiotics, prebiotics, and probiotics within 4 weeks.
• Known allergic history to any Chinese herbal medicines;
• Known pregnancy or lactating.

## 7. Patient visit

### 7.1. Screening visit

Subjects will be invited to come for screening. Information about the study will be explained and subject will sign the informed consent form before screening. Symptom assessment will be done to confirm if the subjects still have residual symptoms of fatigue and more such as dyspnoea and insomnia. Less than 10 ml blood for hematology, liver and renal function tests will be done as safety measures. A deep throat saliva/professional-administered combined nasal and throat swab test for SARS-CoV-2 will be arranged by using the accredited laboratory or Government service.

### 7.2. Baseline randomization visit

At randomization visit, eligible subjects will have medical consultation and assessment by registered Chinese medicine practitioners (CMPs) investigators. Vital sign will be assessed, medical history and concomitant medications will also be recorded. If the subject has a result of not detected or negative result of SARS-Cov-2 RT-PCR test of deep throat saliva/professional-administered combined nasal and throat swab, and negative medical and travel history related to COVID-19 within 72 h, subjects will be instructed to do a pulmonary function test using a designated spirometer and complete the questionnaires; however, performing deep throat saliva/professional-administered combined nasal and throat swab test and pulmonary function test are not mandatory. Subjects will be randomly assigned (in a 1:1 ratio) to receive 8 weeks of either “CRF” granules 23–42 g daily or placebo granules 23–42 g daily. CMP investigators will prescribe and dispense the study treatment accordingly.

### 7.3. Follow-up visit

Subjects will return for follow-up at week 8 (±4 days), and followed by a post-treatment visit at week 12 (Table 1). Symptom assessment and other questionnaires will be done. Blood tests will be repeated as safety measures at week 8. Subjects will be arranged to have another SARS-Cov-2 RT-PCR test of deep throat saliva/professional-administered combined nasal and throat swab, and confirmed to have negative medical and travel history related to COVID-19 within 72 h before the pulmonary function test at week 8. Performing deep throat saliva/professional-administered combined nasal and throat swab test and pulmonary function test are not mandatory. Treatment compliance will be recorded and adverse events will be captured on a patient diary. A direct telephone line will be provided so that subjects can report any adverse events during office hours between scheduled visits. The subjects will be recommended to attend Emergency Department at the nearest hospital beyond office hours if deemed necessary. To monitor compliance, subjects will be asked to bring back all remaining packs of granules for counting. One time free consultation with 1 month Chinese herbal treatment will be provided to all subjects after completion of study to ensure the follow up visit compliance.

**TABLE 1:** Study schedule.

Items	Screening	Treatment period		Post-treatment follow up
	≤7 days to 0 day	Day 0 (baseline)	Week 8 (±4 days)	Week 12 (±4 days)
Informed consent	X			
Eligibility	X	X		
Medical consultation and assessment		X	X	X
Medical history		X		
Concomitant medication		X	X	X
Blood for safety measures	X		X	
Deep throat saliva or nasopharyngeal swab for SARS-Cov-2 RT-PCR test	*X		*X	
AE/SAE assessment			X	X
Questionnaires		X	X	X
Pulmonary function test		^#^X	^#^X	
Administration of study drug		X	X	
	

*Subjects will have deep throat saliva or nasopharyngeal swab for SARS-Cov-2 RT-PCR test within 72 h before follow up visit but it is not mandatory.

^#^Pulmonary function test will be skipped if no deep throat saliva has been done and showed not detected or negative within 72 h.

### 7.4. Study assessments

#### 7.4.1. Fatigue severity score (FSS)

Fatigue severity score is a 9-item scale which measures the severity of fatigue and its effect on a person’s activities and lifestyle in patients with a variety of disorders. Scores range from 9–63; the higher the score, the greater fatigue severity (23).

#### 7.4.2. Self-reported COVID-19 long term symptom assessment

For the symptom questionnaire, participants will be asked to report newly occurring and persistent symptoms, or any symptoms worse than before COVID-19 development. The symptom assessment has 5 scale, from none to very severe.

#### 7.4.3. The modified British medical research council (mMRC) dyspnoea scale

The mMRC scale is a five-category scale to characterize the level of dyspnoea with physical activity in which higher scores correspond with increased dyspnoea (6).

#### 7.4.4. EuroQol five-dimension five-level (EQ-5D-5L)

The EQ-5D-5L is a validated questionnaire to evaluate patient quality of life by assessment of the following five domains: mobility, self-care, usual activities, pain or discomfort, and anxiety or depression. Categorization within each factor is divided into five-levels that range from no problems to extreme problems (6). EuroQol Visual Analogue Scale (EQ-VAS) is a patient’s subjective assessment of generic health ranging from 0 (worst imaginable health) to 100 (best imaginable health) before COVID-19 and at the time of the visit, with higher scores representing better subjective health experience. A difference of 10 points defined worsened quality of life (6).

#### 7.4.5. Pulmonary function test device

FEV1, FVC, and FEV1/FVC ratio will be measured with the Air Next Spirometer (NuvoAir, Sweden) in combination with the mobile coaching system. Air Next is a novel spirometer that connects *via* bluetooth to a smartphone application, permitting patients to monitoring lung function at home. It has a turbine mechanism (Flow Mir) to perform measurements inside the disposable single use nozzles. To perform spirometry, the user exhales air into the turbine. This air turns a motor, and the device registers the speed of the rotor, adapts it, and transfers the data to the smartphone application (24). The higher of FEV1/FVC ratio, the healthier of subjects.

#### 7.4.6. Blood test

Venous blood samples will be collected from all participants for hematology, liver and renal function tests as safety measures.

#### 7.4.7. Subject withdrawal

A subject must be withdrawn from the study if he/she withdraws consent. Subjects who (1) experience adverse events, or (2) have pre-existing violation of entry criteria may remain in the study unless the investigator determines that it is not in the subject’s best interest to continue. The specific reason for withdrawal should be indicated.

Subjects who have withdrawn from the study will be invited to follow up at the last study visit i.e., 8 weeks after the randomization visit to detect any delayed clinical events.

## 8. Study intervention

### 8.1. Study treatments

The core study treatment, COVID Rehab Formula “CRF” is prepared in the form of mixed LJZD and SSMDD concentrated herbal granules with variations.

Essentially, the “CRF” consists of Ginseng Radix Et Rhizoma (renshen) 8 g, Atractylodis Macrocephalae (baizhu) 7.2 g, Rhizoma Poria (fuling) 7.2 g, Glycyrrhizae Radix Et Rhizoma Praeparata Cum Melle (zhigancao) 4.8 g, Glycyrrhizae Radix Et Rhizoma (gancao) 4 g, Pinelliae Rhizoma Praeparatum (fabanxia) 9.6 g, Citri Reticulatae Pericarpium (chenpi)7.2 g, Adenophorae Radix (nanshashen) 8 g, Polygonati Odorati Rhizoma (yuzhu) 8 g, Mori Folium (sangye) 4.8 g, Ophiopogonis Radix (maidong) 8 g, Lablab Semen Album (baibiandou) 8 g, and Radix Trichosanthis Radix (tianhuafen) 8 g.

For study subjects with the following symptoms, specified Chinese medicine will be added; fatigue: Astragali Radix (huangqi) 15 g; dyspnea or cough with sputum: Trichosanthis Pericarpium (gualoupi) 10 g, Fritillariae Thunbergii Bulbus (zhebeimu) 10 g, Armeniacae Semen Amarum (kuxingren) 10 g; sleep disturbance: Ziziphi Spinosae Semen (suanzaoren) 10 g, Indian Bread with Pine (fushen) 10 g and Margaritifera Concha (zhenzhumu) 10 g; loose stool: Plantaginis Semen (cheqianzi) 10 g, Artemisiae Scopariae Herba (mianyinchen) 15 g, and Coicis Semen (yiyiren) 10 g.

Granules will be used with the dosage equivalent to the raw herbs according to the manufacturer. One daily dose of which (weight 23–42 g) will be dissolved in hot water and administered while it is lukewarm. Study subjects will take 2 times daily, 1–2 packs in the morning and the other in the evening after meal. The placebo granules, made of starch and caramel with similar appearance, smell and taste to the CRF but contain no active constituents, will be taken by the patients in the placebo group in the same way as those in the treatment group.

Both treatment herbal and placebo granules will be produced by a manufacturer with a Good Manufacturing Practice (GMP) certificate.

### 8.2. Prohibited drugs

Non-steroidal anti-inflammatory drugs (NSAIDs), steroids and antibiotics are prohibited. Chinese medical treatments which are used to treat those residual symptoms are prohibited during the study period.

### 8.3. Treatment compliance

Treatment compliance is assessed by the number of doses taken during study period by subjects’ self-report and research personnel’s counting of returned packs of granules.

### 8.4. Randomization and blinding

A computer-generated randomization schedule is used to assign subjects to the treatment sequences. Concealment of allocation will be ascertained by an independent research staff member, and identically designed treatment packs will be used for study drugs.

This is a double-blind randomized controlled trial. The random allocations will be put into opaque envelops with sequential study numbers. Two sets of the envelope will be prepared, with one set for randomization at the site, and another set for storage in the investigator’s office for emergency un-blinding.

## 9. Possible risks and adverse event reporting

### 9.1. Possible risks and discomfort

In general, there will be minimal discomfort in blood taking and the risk include bleeding, infection, bruising, and feeling lightheaded. Besides, no serious adverse event has been reported in both decoction (25, 26) and their variations such as Indian Bread with Pine, Ziziphi Spinosae Semen and Margaritifera Conch (27).

There is mild discomfort of taking Shashen Maidong Decoction, Plantaginis Semen and Artemisiae Scopariae Herba including feeling of abdominal discomfort, diarrhea, abdominal distension, nausea, and vomiting as well as loss of appetite (28, 29). On the other hand, studies show that Fritillariae Thunbergii Bulbus may cause toothache, hand numbness and induce allergy reaction, but those can be relieved after treatment (30). Astragali Radix are found to induce allergy reaction, painful sensation on limbs, headache, insomnia and abdominal discomfort, but those may due to overdose usage (over 30 g) and using injection dosage form (31), while Trichosanthis Pericarpium are found to cause allergy reaction and abdominal discomfort, those are possibly by using injection dosage form (32). However, there is no documented evidences about possible risks and discomfort for Coicis Semen and Armeniacae Semen Amarum. Moreover, according to reports of interaction of CM and Herbal Medicine(s) with Anti-cancer Drugs forms, published by The University of Hong Kong, Department of Pharmacology and Pharmacy, there is no evidence for AE or harmful interaction between COVID-19 Rehabilitation Formula (CRF) and western medicine.

### 9.2. Adverse events

An adverse event is any undesirable medical event occurring in the subject within the trial period, whether or not it is related to the study intervention. No serious adverse event has been reported of taking COVID-19 Rehabilitation Formula (CRF). The adverse reaction will be recorded and the treatment will be suspended when severe adverse reaction occurs. The assessment of adverse event will be recorded according to CTCAE 4.0 which is a standard assessment tool.

### 9.3. Serious adverse events

A serious adverse event is an adverse event that results in one of the following outcomes:

• Death.
• Life-threatening.
• In-patient hospitalization or prolongation of existing hospitalization.
• A persistent or significant disability or incapacity.
• A congenital anomaly or birth defect.

The definitions of causal relationship to study intervention are the same as those for adverse events. A standard serious adverse event form will be used (provided by The Joint Chinese University of Hong Kong---New Territories East Cluster Clinical Research Ethics Committee [Joint CUHK-NTEC CREC] at)^2^ to report the events within 24 h after acknowledgement.

## 10. Sample size and statistical methods

### 10.1. Sample size calculation

The primary outcome is Fatigue Severity Score (FSS). There is no any previous study on the Chinese medicine treatment of COVID-19 measured by FSS. The clinically meaningful improvement in FSS was assumed to be a drop of 9 out of 63 based on previous studies (33, 34). Based on the standard deviation of symptom score of residual fatigue in this study on COVID-19 recovered patients (33, 35), we assumed standard deviation estimates of 12. To detect a clinically meaningful difference of 9 points in FSS, sample size of 58 was estimated based on standard deviation estimates of 12 at an alpha of 0.05 and power of 0.8. It should be noted that these estimates are considered vague approximations, and 58 participants is a conservative estimate to detect a statistically significant result of change in FSS. To compensate a possible drop-out rate of 15%, totally 68 patients will be needed, with 34 in each group.

### 10.2. Statistical analysis

Baseline data (gender, age, and vital signs) will be descriptively summarized. Differences of measurement data between the groups will be assessed using *t*-test for normally distributed continuous variables and Wilcoxon signed rank test for non-normally distributed variables. Measurement data of different groups in each visit will be reported as mean ± standard deviation (SD). Intra-group comparisons between baseline and each visit will be conducted by using paired *t*-test (or Wilcoxon signed rank test). Comparisons between groups will be conducted by using an analysis of variance (ANOVA), with other confounding factors like multicenter character conducting the covariate analysis. Statistical analysis for the data which do not meet above conditions (e.g., non-normal) will be conducted with the use of non-parametric test. For laboratory parameters, Spearman’s coefficient correlation has been used for comparison with different parameters and one way ANOVA test for significant correlation between groups. Study flow chart and graphs will be presented over different study times.

All *p*-values and 95 percent confidence intervals are two-sided, and *p* < 0.05 is considered statistically significant. Statistical analyses will be carried out exclusively by an independent statistician. All analyses will be performed by using SPSS, version 24 (SPSS).

## 11. Ethics consideration

Application for ethical approval have been sought from Joint CUHK-NTEC CREC before the initiation of this study. All important protocol modifications are approved by the said CREC before implementation (except in emergency situations where immediate hazards to the subjects have to be eliminated) and communicated to investigators, research personnel and trial participants. Participants are informed of our measures to ensure the confidentiality of all information and that data are maintained anonymous. Investigators/research personnel obtain informed consents from subjects and consent forms are signed by the subjects before the study. All information are encrypted and only the involved investigators can access. Password is required to access the data. Participants are free to withdraw at any time without giving a reason or punishment. The personal data of the subjects will only be kept for 10 years and will be destroyed afterward.

## 12. Clinical trial insurance

Clinical trial insurance will be purchased according to the university policy.

## 13. Quality assurance

### 13.1. Data management

Only the study team and the PI have access right to the database using their login user names and passwords. Subjects’ information is kept anonymous. They are identified by their study numbers to maintain subjects’ confidentiality. The final dataset of the study will be available from the corresponding author on reasonable request.

### 13.2. Auditing and inspection

The Clinical Research Management Office at The Chinese University of Hong Kong or an external auditor will perform auditing or inspection to determine whether research activities are conducted according to the protocol, Good Clinical Practice (GCP) and guidelines of the International Conference on Harmonization (ICH).

## 14. Dissemination policy

We will communicate the results mainly through scientific publications and/or press conferences. No publication restrictions are planned. Authorship will be determined based on the criteria adopted by the International Committee of Medical Journal Editors.

## Ethics statement

Approval (reference number: 2021.125-T) has been obtained from The Joint Chinese University of Hong Kong—New Territories East Cluster Clinical Research Ethics Committee. Informed consent will be signed before subject enrolment. The results will be submitted to peer-reviewed journals and presented at academic conferences.

## Author contributions

ZL: conceptualization and funding acquisition. JC, CL, TS, PC, CS, and ML: methodology. CS, WM, KL, and SL: investigation. CL, CS, CC, and KC: project administration. ZL, JC, and KC: supervision. CL, TS, PC, CS, and ML: writing — original draft. ZL, CS, TS, and HZ: writing — review and editing. All authors contributed to the article and approved the submitted version.

## Footnotes

1
https://www.chp.gov.hk/en/resources/346/index.html

2
https://www.crec.cuhk.edu.hk/wp-content/uploads/2022/09/Standard-SAE-Report-Form_Version-2-dated-09-May-2017.pdf
